# Pneumonitis after Stereotactic Thoracic Radioimmunotherapy with Checkpoint Inhibitors: Exploration of the Dose–Volume–Effect Correlation

**DOI:** 10.3390/cancers14122948

**Published:** 2022-06-15

**Authors:** Kim Melanie Kraus, Caroline Bauer, Benedikt Feuerecker, Julius Clemens Fischer, Kai Joachim Borm, Denise Bernhardt, Stephanie Elisabeth Combs

**Affiliations:** 1 Department of Radiation Oncology, School of Medicine and Klinikum Rechts der Isar, Technical University of Munich (TUM), 81675 Munich, Germany; caroline.bauer@mri.tum.de (C.B.); julius.fischer@tum.de (J.C.F.); kai.borm@mri.tum.de (K.J.B.); denise.bernhardt@tum.de (D.B.); stephanie.combs@tum.de (S.E.C.); 2 Institute of Radiation Medicine (IRM), Helmholtz Zentrum München (HMGU) GmbH, German Research Center for Environmental Health, 85764 Neuherberg, Germany; 3 Partner Site Munich and German Cancer Research Center (DKFZ), Heidelberg, German Cancer Consortium (DKTK), 80336 Munich, Germany; benedikt.feuerecker@tum.de; 4 Department of Radiology, School of Medicine and Klinikum Rechts der Isar, Technical University of Munich (TUM), 81675 München, Germany; 5 Department of Radiology, University Hospital Munich, LMU Munich, 81377 Munich, Germany

**Keywords:** radioimmunotherapy (RIT), checkpoint inhibitors, stereotactic body radiation therapy (SBRT), lung cancer

## Abstract

**Simple Summary:**

Stereotactic body radiation therapy (SBRT) is widely applied for treatment of early stage lung cancer and pulmonary metastases. Modern immune checkpoint blockade (ICB) is progressively used in cancer treatment. Pneumonitis is a relevant side effect of both thoracic SBRT and ICB. Currently, it remains unclear whether we can presume the same radiation dose–volume–effect correlations and dose constraints for safe application of SBRT + ICB. We present a dose–volume–effect correlation analysis method using pneumonitis contours and dose–volume histograms (DVH). We showed dosimetric differences for pneumonitis volumes between SBRT + ICB and SBRT alone. We found a large extent of pneumonitis, even bilateral and apart from the radiation field for combined SBRT + ICB. We noticed a shift in pneumonitis DVHs towards lower doses and a trend towards decreased areas under the curve (AUC) for SBRT + ICB. This provides a direction for re-evaluation and potential adaptation of lung dose constraints for combined SBRT and ICB.

**Abstract:**

Thoracic stereotactic body radiation therapy (SBRT) is extensively used in combination with immune checkpoint blockade (ICB). While current evidence suggests that the occurrence of pneumonitis as a side effect of both treatments is not enhanced for the combination, the dose–volume correlation remains unclear. We investigate dose–volume–effect correlations for pneumonitis after combined SBRT + ICB. We analyzed patient clinical characteristics and dosimetric data for 42 data sets for thoracic SBRT with ICB treatment (13) and without (29). Dose volumes were converted into 2 Gy equivalent doses (EQD2), allowing for dosimetric comparison of different fractionation regimes. Pneumonitis volumes were delineated and corresponding DVHs were analyzed. We noticed a shift towards lower doses for combined SBRT + ICB treatment, supported by a trend of smaller areas under the curve (AUC) for SBRT+ ICB (median AUC 1337.37 vs. 5799.10, *p* = 0.317). We present a DVH-based dose–volume–effect correlation method and observed large pneumonitis volumes, even with bilateral extent in the SBRT + ICB group. We conclude that further studies using this method with enhanced statistical power are needed to clarify whether adjustments of the radiation dose constraints are required to better estimate risks of pneumonitis after the combination of SBRT and ICB.

## 1. Introduction

In the 21 century, immunotherapy with checkpoint inhibitors has revolutionized the treatment of lung cancer and metastatic disease for numerous cancers with improved clinical outcome [1,2]. Even better results are expected by combination of radiation therapy with immunotherapy by synergistic enhancement of immunological mechanisms [3]. While radiation is cytotoxic, it also impacts the tumor itself, e.g., by upregulation of Programmed Cell Death-Ligand 1 (PD-L1) and Programmed Cell Death-1 (PD-1) [4], and its microenvironment by increased anti-tumor response mechanisms [5,6,7]. Thus, evidence for combined RIT, especially with ICBs, for clinical application for primary lung tumors and pulmonary metastases is growing, with a variety of ongoing studies [8] showing improved clinical outcome [9,10,11,12,13]. However, to date it is still debated which radiotherapy (RT) regimens (e.g., dose per fraction) are optimal to stimulate synergies with ICB [7]. SBRT is an elegant treatment alternative for early stage lung cancer for medically inoperable patients or those who refuse surgery as well as for pulmonary metastases with excellent local control rates [14,15,16,17,18,19,20]. Furthermore, the combination of SBRT and ICB treatment has been investigated with beneficial clinical outcome [21,22,23] and a multitude of clinical trials is currently ongoing [8,24,25]. In current clinical guidelines, for early stage lung cancer, usually adjuvant chemotherapy is administered for stage II and III [26]; ICB **can be** considered for adjuvant treatment in stage IIA, IIB and IIIA, and **is recommended** for node-positive **lung** cancer after **definitive** chemo**radiation** [15]. Secondary lung cancer can also be treated with surgery, systemic agents and radiotherapy, usually depending on the primary tumor and the number and spread of metastases [27].

Pneumonitis is a relevant and dose-limiting adverse event for RT with a wide range of occurrence rates, between 9% to 28%, depending on the dose, irradiated volume and prior interstitial lung disease [28]. For monotherapy with ICBs of Non-Small Cell Lung Cancer (NSCLC), pneumonitis occurs in less than 5% [29,30] and in about 10% for combination ICB therapy [30]. The phase III PACIFIC trial investigating durvalumab (anti-PD-L1) after fractionated radiochemotherapy for NSCLC showed enhanced all-grade pneumonitis rates in up to 34% in the durvalumab group compared to 25% in the placebo arm. In the KEYNOTE trial, analyzing subsequent immunotherapy after RT, all-grade pulmonary toxicity was significantly higher in the RT + ICB group (13% vs. 1%, *p* = 0.046) [12]. Furthermore, a large database analysis revealed numerically increased rates of pulmonary adverse events in patients who received ICB within 90 days prior to RT. However, details of the RT regimens were not part of the study [31]. When it comes to SBRT, the majority of studies on combination of SBRT and ICB suggest increased total rates of pulmonary toxicity after combination of SBRT and ICB; however, combination therapy was not found to enhance high-grade side effects [23,32,33,34,35,36]. The current evidence is based on studies with a multitude of dose regimes ranging from 30 Gy to 50 Gy in 3 to 5 fractions. Thus, to date, the optimal dose fractionation and treatment sequence to reduce pulmonary side effects remain unclear.

Since pneumonitis can originate from multifactorial mechanisms under combined RIT, there is reason to re-consider established and well-known dose–volume–effect correlations for SBRT combined with ICB treatment. While the majority of current studies focus on the clinical outcome, including toxicity rates, there are currently very limited data investigating the potentially required adaptation of dose constraints. However, for clinical application of RIT, these data is of major relevance.

In this study, we investigate dosimetric parameters from pneumonitis DVHs. We aim to find out whether immunotherapy in combination with SBRT affects the development and extension of treatment-related pneumonitis. We approach this by revealing the potential correlation between radiation dose and the corresponding extension of pneumonitis for combined thoracic SBRT and ICB treatment compared to SBRT alone.

## 2. Materials and Methods

### 2.1. Clinical Patient Data

We collected 42 data sets from patients who received thoracic SBRT at our institute. Thirteen patients received additional immunotherapy with ICB including either PD-1 or PD-L1 inhibitors within a time frame of 50 days around RT for primary lung cancer (7) or pulmonary metastases (6). One patient received a combination of PD-1 and cytotoxic T-lymphocyte-associated Protein 4 (CTLA-4) antibody. A total of 29 patients received SBRT alone, out of which 11 were treated for secondary cancer. All patients in the SBRT + ICB group had peripherally located tumors and three patients in the SBRT group had centrally located tumors.

In the SBRT + ICB group, seven patients received pembrolizumab and six patients received nivolumab. Nine patients received ICB within a time interval of less than 20 days around RT. Five patients received ICB prior and after RT, two patients received simultaneous ICB, in four cases ICB was administered only prior to RT, and two patients received ICB after RT. Twenty-one patients received chemotherapy prior, after or simultaneously to RT. Agents covered a wide range according to the treatment indication; however, from those 21 patient cases, in only four cases chemotherapy was administered within a window of less than 50 days around RT. The administered drugs comprised two platinum-based agents, pemetrexed and dacarbazine.

The radiotherapy fractionation schemes widely varied individually from single doses of 4 Gy to 15 Gy and total doses of 21 Gy up to 60 Gy. In the SBRT + ICB group, two patients received 50 Gy in 5 Gy single fractions, three patients received 37.5 Gy in 12.5 Gy single fractions, three patients 60 Gy in 7.5 Gy single fractions, four patients 45 Gy in 15 Gy single fractions and one patient received 30 Gy in 5 Gy single fractions. Three tumors in the SBRT group were centrally located. One of them was a metastasis treated with 40 Gy in 8 Gy fractions and two of them were primary lung tumors treated with a dose of 60 Gy in 7.5 Gy fractions. In the ICB + SBRT group, seven patients received a biologically effective dose with α/β = 10 depicted as BED_10_ ≥ 100 Gy and 11 in the SBRT group. SBRT was administered as intensity-modulated radiotherapy and in the majority of cases it was delivered as a volumetric rotational therapy. In fifteen cases, patients received additional prior thoracic RT; seven in the SBRT + ICB group and two of them at the ipsilateral side as the investigated location, which were given 364 and 628 days prior to the investigated RT treatment course. Eight patient cases of the SBRT-only group received prior thoracic RT and five of them were located at the same side as the investigated case. The shortest time between prior ipsilateral RT and the investigated RT treatment course was 292 days, and the majority happened several years before the studied therapy.

Clinical patient characteristics, including occurrences of pneumonitis, were extracted from patient records and are depicted in Table 1. Pneumonitis was evaluated according to the Common Terminology Criteria for Adverse Events (CTCAE) v5.0 [37]. Descriptive statistical analysis was performed using IBM SPSS Statistics 25. Univariate analysis and analysis of significance was performed using chi-squared tests or Mann–Withney U tests, with a significance level of 0.05.

### 2.2. Dosimetric Data

Based on the data from the clinical patient files regarding the occurrence of pneumonitis, we identified the corresponding follow-up computed tomography (CT) scans, showing the radiological features of pneumonitis for the first time. Since pneumonitis can originate from RT as well as ICB treatment, radiographic findings are various. Most commonly described features include cryptogenic organizing pneumonia (COP), with ground-glass or consolidative opacities; and nonspecific interstitial pneumonia (NSIP), with ground-glass opacities and reticular opacities [30,38,39,40]. Based on these findings, the pneumonitis-comprising volume was delineated for each patient using the treatment planning software Eclipse 15.6 and 16.0 (Varian Medical Systems, Palo Alto, CA, USA), checked by a specialist in nuclear medicine with experience in radiological diagnostic imaging. The contours were transformed to the RT planning CT using rigid and subsequent deformable image registration with a modified “demons” algorithm [41]. Additional contours, required for further processing, such as the gross tumor volume (GTV) and the lung were also delineated. The contour as well as dose data were extracted from the treatment-planning system. A MATLAB-based (MATLAB R2019b, The MathWorks Inc., Natick, MA, USA) software tool was used to convert the clinically applied doses to 2 Gy fractions equivalent doses (EQD2) on a voxel basis in order to compare various fractionation schemes using a linear quadratic model (LQM) [42]. We assumed an α/β ratio of three and ten for normal lung tissue and the lung tumor volume, respectively [43]. Dosimetric analysis was performed using the open-source platform 3D Slicer [44] and the RT toolkit [45]. In case of multiple thoracic radiation within a time interval of six months, we used sum doses. We report the relevant dose and volume parameters as the mean with standard deviation (SD) and median with range. We performed univariate analysis and analysis of significance was performed using chi-squared tests and Fisher’s exact tests for categorial variables. For numeric data, due to the small sample size and partially missing normal distribution, we applied Mann–Whitney U tests, with a significance level of 0.05.

## 3. Results

Our results indicate a trend towards altered correlation between dose and the extension of pneumonitis for combined SBRT and ICB treatment compared to SBRT alone.

We exploratory investigated 13 and 29 patient cases with SBRT + ICB treatment and SBRT alone, respectively. Comparing extensions and doses of the pneumonitis volumes, we found numerically increased mean pneumonitis volumes (177.64 cm^3^ vs. 120.40 cm^3^). However, a trend of smaller, even not significant, median values (68.82 cm^3^ vs. 99.43 cm^3^, *p* = 0.641) was noticeable. Median planning target volumes (PTV) were numerically smaller for SBRT + ICB (26.30 cm^3^ vs. 46.70 cm^3^, *p* = 0.317). At the same time, we found a trend to smaller median EQD2 (13.32 Gy vs. 57.97 Gy, *p* = 0.317) and noticed smaller median pneumonitis volumes receiving at least 20 Gy (V20) (33.85 cm^3^ vs. 68.22 cm^3^, *p* = 0.039) for the SBRT + ICB group compared to SBRT alone. There was a trend of delayed onset of pneumonitis for the SBRT + ICB group (71 days vs. 47 days after RT, *p* = 0.174). Details are listed in Table 1.

For three patients, pneumonitis even extended bilaterally despite a low median of the mean lung dose (MLD) of 5.31 Gy for the SBRT + ICB group, which was higher in the SBRT group (7.13 Gy) (see Table 1). Only one patient in the solely SBRT group showed bilateral extent, which can be attributed to bilateral SBRT.

**Figure 1:**
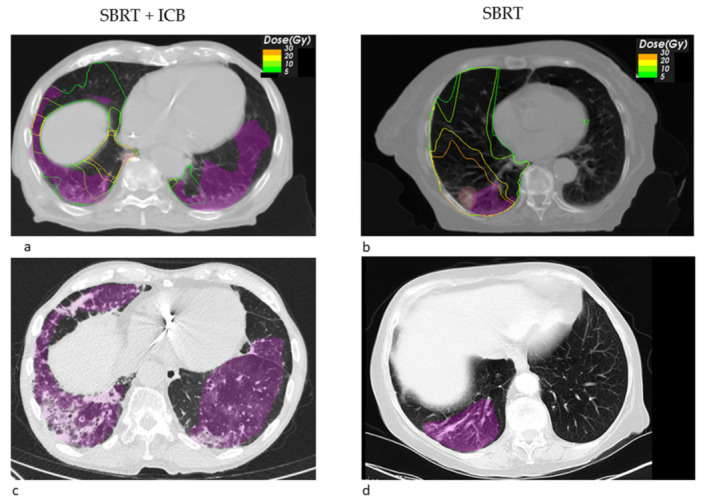
Transversal radiotherapy (RT) planning CT slices showing the EQD2 isodoses and pneumonitis contours (magenta) and GTV contours (light red) for a SBRT + ICB case (**a**) and for a SBRT case (**b**). Exemplary transversal slices from the follow up CT showing the pneumonitis and its contours (magenta) for the SBRT + ICB case (**c**) and the SBRT case (**d**).

**Figure 2:**
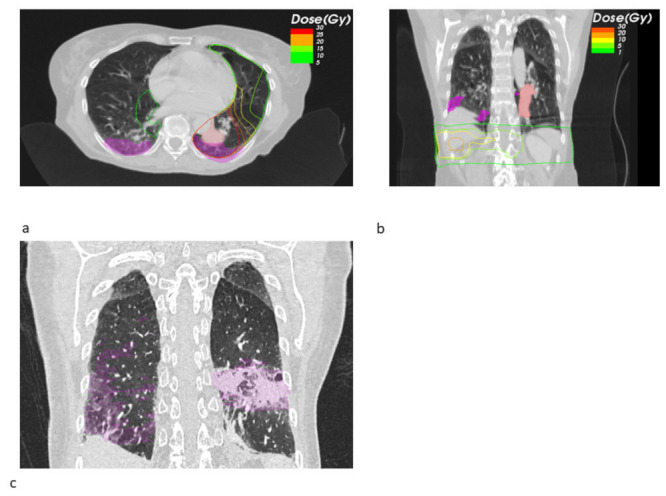
Transversal radiotherapy (RT) planning CT slices showing the sum EQD2 isodoses and pneumonitis contours (magenta) and GTV contours (light red) for a SBRT+ ICB case (**a**). This patient received simultaneous liver metastasis SBRT. The isodoses of the original treatment plan show a negligible lung dose (**b**). Figure (**c**) shows an exemplary sagittal slice from the follow-up CT showing the bilateral pneumonitis and its contours (magenta).

**Figure 3:**
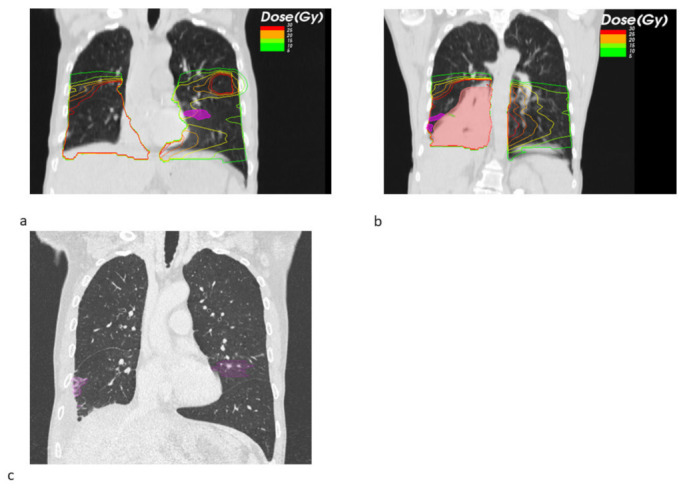
Sagittal radiotherapy (RT) planning CT slices showing the sum EQD2 isodoses and pneumonitis contours (magenta) for a SBRT+ ICB case (**a**). The same patient received simultaneous contralateral hypofractionated mediastinal RT with original isodoses revealing a relevant lung dose (**b**). Exemplary sagittal slice from the follow-up CT showing the bilateral pneumonitis and its contours (magenta) (**c**).

Looking at the dosimetric results in more detail, we observed a trend towards a decreased fraction of the pneumonitis volume receiving a high dose of EQD2 > 20 Gy SBRT + ICB (22.26% vs. 71.14%, *p* = 0.549), whereas the mean low dose fraction with EQD2 < 10 Gy was numerically larger (39.02% vs. 26.99%). Figure 1 shows the pneumonitis extensions and EQD isodoses for two exemplary cases from both groups. For the depicted SBRT + ICB case, the pneumonitis affects both lungs with a rather small single-sided GTV distant from the resulting radiological changes. For another patient, who received SBRT with 45 Gy in 3 fractions to a small left-sided tumor, we also found bilateral extent of pneumonitis, as shown in Figure 2. This patient received additional SBRT for liver metastasis at the same time with the same dose fractionation regime; however, the extent of pneumonitis does not correlate with the dose distribution. For dosimetric analysis, we used sum doses to prevent overestimation of the effect attributed to the lung SBRT. A third patient also showed the bilateral extent of pneumonitis. This patient received hypofractionated, contralateral mediastinal RT with 45 Gy in 15 fractions a month prior to SBRT. Since the extent of the pneumonitis volume in the contralateral right lung is more likely caused by the irradiation of the right side, we considered only the pneumonitis contour from the left SBRT side for analysis. However, to not overestimate the dosimetric effect of SBRT alone, we used the sum EQD2, as shown in Figure 3a, for dosimetric evaluation as well.

**Figure 4:**
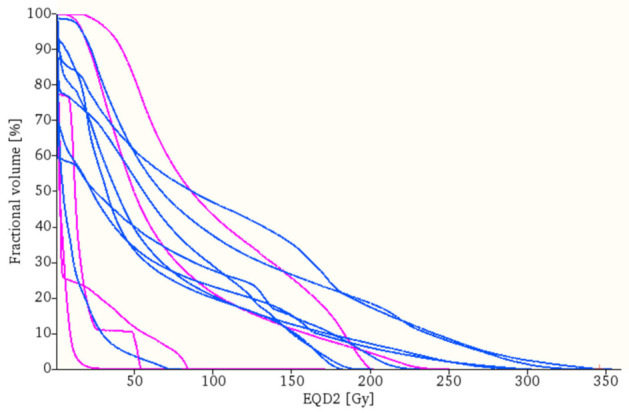
Dose–volume histograms (DVHs) for pneumonitis volume for SBRT + ICB (pink) and SBRT (blue). Doses are indicated in 2 Gy equivalent doses (EQD2). SBRT pneumonitis DVHs are shifted towards higher doses.

Analyzing the DVHs, we noticed a trend towards lower doses for combined SBRT + ICB treatment, as can be seen in Figure 4. This trend was supported by a numerical difference in the area under the curve (AUC), which was smaller for SBRT + ICB compared to SBRT alone (median AUC 1337.37 vs. 5799.10, *p* = 0.317), even though the data were not significant.

Patient characteristics are depicted in Table 1. All but two parameters did not significantly differ between the groups. However, the number of patients suffering from additional pulmonary diseases was smaller for the combined SBRT+ ICB cohort (1 (7.7%) vs. 15 (51.7%), *p* = 0.006). In total, three patients with pneumonitis had known lung disease (chronic obstructive pulmonary disease (COPD) or emphysema), from whom none received ICB treatment. The number of active or former smokers was comparable in both groups (8 (61.5%) vs. 17 (58.6%), *p* = 0.595).

The fractionation schemes varied widely. However, in all of the different RT groups, one or even two patients developed pneumonitis, except for the last indicated fractionation scheme with a total dose of 30 Gy in 5 Gy fractions. One patient received a combination of PD-1 and CTLA-4 antibody and developed pneumonitis 145 days after RT.

Statistically significant more patients in the SBRT + ICB treatment group had a history of chemotherapy (10 (76.9%) vs. 11 (37.9%), *p* = 0.019). Prior thoracic RT was numerically enhanced in the SBRT + ICB group (7 (53.8%) vs. 8 (27.6%), *p* = 0.101), from whom six developed pneumonitis (three with ICB and three without ICB treatment).

## 4. Discussion

Our results indicate altered dose–volume–effect correlations regarding the development of pneumonitis for combined treatment with SBRT and ICB compared to SBRT alone. We demonstrate the applicability of a method based on DVH analysis of pneumonitis volumes. We observed a trend of numerically enlarged pneumonitis volumes developing within lower radiation dose fields. These exploratory findings might trigger further re-evaluation of potential changes in dose prescription and dose constraints for the lung based on extended patient cohorts.

The majority of existing data in the literature investigated the incidence of pneumonitis after sequential or concurrent ICB treatment with RT and revealed increased all-grade lung injury with no increased high-grade toxicity [23,46,47] an thus is regarded to be safe. A multitude of studies analyzing SBRT and ICB therapy is currently ongoing and has been gathered in several reviews [7,25,48]. However, limited data suggest that the risk for pneumonitis and the resulting consequences with regard to treatment discontinuation might be underestimated [49,50,51] and the dose–volume relationships regarding the development of pneumonitis remain unclear.

Watanabe et al. [52] used a similar approach to discover factors that predict pneumonitis above grade 2 and found V5 to V50 to be significantly smaller for grade 2 pneumonitis compared to grade 1 after chemoradiotherapy. However, to our knowledge, the study presented here is the first that applies this method to examine dosimetric correlations between the dose and pneumonitis volumes for combined SBRT + ICI in comparison to SBRT alone. We noticed a trend towards numerically large pneumonitis volumes for SBRT + ICB treatment, not restricted to high dose (EQD2 > 20 Gy) radiation fields and even with extent to the contralateral lung in three cases and a late median onset (71 days after RT). Due to the patterns of pneumonitis, the volumetric data can potentially overestimate the extent of the involved lung tissue. Data in the literature on the radiographic patterns and correlation to radiation dose for the combination of RT and ICB treatment are sparse [53]. Radiation-induced pneumonitis is commonly restricted to the radiation field [54] and appears within three to 12 weeks after radiation [54]. For larger fraction doses, V20  >  10% and a mean lung dose  >  8 Gy are associated with an increased risk of pneumonitis ≥ grade 2 [55,56,57,58], and high MLD even impacts survival [59]. In this study, the median MLD_EQD2_ over all patient cases was below 8 Gy for both groups and even lower for the SBRT + ICB group (5.31 Gy vs. 7.13 Gy, *p* = 0.641). Not surprisingly, statistical significance is missing due to the sparse number of data sets. V20_total lung_ was below 8% for both groups. Thus, solely radiation-induced pneumonitis seems unlikely. Immune-related manifestation of pneumonitis comprises a variety of radiographic patterns with a variable, multifocal and bilateral extent [60,61], with a mean onset after 3 months post immunotherapy [38,61]. The incidence varies also with agent and is considered to be 3–5% for PD-1 and PD-L1 and 1% for CTLA-4 inhibitors [60]. Two small case studies support the finding of extended pneumonitis volumes and late onset of pneumonitis. Schoenfeld et al. [51] reported a patient case receiving fractionated axillary RT prior to ICB treatment who developed pneumonitis five months after RT with extension also apart from the radiation field but restricted to the ipsilateral lung despite a low V20 of 8% of the total lung. Manapov et al. [49] reported three patient cases with pneumonitis after thoracic RT and subsequent ICB therapy with nivolumab and found parenchyma changes in the radiation field that received 15 to 20 Gy. However, for both studies, low mean lung doses and late onset (5 months and 167 days after RT) assume an interplay between radiation-induced and immunogenic origin of pneumonitis and a probable enhancement of immune-stimulating effects by radiation.

Known potential risk factors, such as previous RT, radiation dose and volume, chemotherapy and pulmonary comorbidity, varied among our patient cohort. Clearly, the retrospective study character and the small sample size of the here-presented data can only provide exploratory observations and trends without statistical significance. From the patients with pneumonitis, three had prior known lung disease and none of them received ICB treatment, which forms a statement against pulmonary disease as a confounder to the increased pneumonitis volumes in the SBRT + ICB groups. About 50% and 30% received prior thoracic RT in the SBRT + ICB group and the SBRT group, respectively. From all fourteen patients with pneumonitis, six patients received prior thoracic RT, equally distributed between both study groups. Thus, a history of prior thoracic RT can contribute to the increased likelihood of pneumonitis development for the RIT group. Furthermore, for SBRT of early stage lung cancer, the patient often is medically inoperable or refuses surgery. Being medically inoperable can often be associated with a compromised pulmonary function, also due to interstitial lung disease. This could also contribute to enhance appearance of pneumonitis in these patients. We also presented two cases in the SBRT + ICI group with simultaneous (within less than a week) or shortly sequential contralateral thoracic RT, with a bilateral extent of the pneumonitis. In one case, we could demonstrate the spatial independency of the resulting pneumonitis and the RT contralateral to the SBRT. For both, we evaluated the sum doses to not overestimate the effect of the SBRT dose. Fractionation schemes also widely varied, which, due to dose–volume–effect correlations [62], could impact inter-individual comparability. This issue has been addressed by conversion of doses to 2 Gy fractions equivalent doses.

Due to the diversity of indications, also the treatment sequence was different. From the thirteen patients receiving ICB treatment, five had ICB therapy prior to RT, two concomitant, four prior and after RT and two only after RT. Importantly, the median time between ICB and SBRT was 8.5 days and only patients that received ICB treatment within a maximum of 50 days around RT were considered for this study. Due to the sample size and diversity, we cannot draw definitive conclusions regarding the treatment sequence. However, the optimal sequence is a crucial topic of current research [8,25]. Further studies will be needed to address this point. While immune stimulation by radiation favors RT prior to immunotherapy, the same effects can enhance normal tissue toxicity. Von Reibnitz et al. [63] compared concurrent and sequential (within one month) SBRT and ICB treatment and could not find significant toxicity differences between the two groups.

In summary, this study shows an exploratory analysis of the dose–volume–effect correlation for the development of pneumonitis after combined SBRT and ICB treatment compared to SBRT. We found a trend towards large extents of pneumonitis also apart from the radiation field and despite low radiation doses and a shift of the DVHs towards lower doses for combined SBRT + ICB.

## 5. Conclusions

We present a DVH-based dose–volume–effect correlation analysis method for pneumonitis. We discovered a trend towards lower doses and extended pneumonitis volumes after SBRT + ICB compared to SBRT alone. The statistical significance of these findings has to be confirmed by studies with larger patient cohorts. The data presented here point in the direction of a re-evaluation of the current lung dose constraints applied in combined SBRT and ICB therapy.

## Figures and Tables

**Table 1:** Patient characteristics and dosimetric data. Doses are reported in 2 Gy fractions equivalent doses (EQD2).

Patient Characteristics						
		SBRT+ICB	SBRT+ICB (%)	SBRT	SBRT (%)	*p*-Value
No. of patients		13	100.0	29	100.0	
No. of females		7	53.8	15	51.7	0.582
No. of males		6	46.2	14	48.3	
Median age (a)		69		74		0.282
Pulmonary co-morbidity		1	7.7	15	51.7	0.006
Active or former smokers		8	61.5	17	58.6	0.595
No. of patients with lung metastases		6	46.2	11	37.9	0.369
No. of patients with primary lung tumors		7	53.8	14	48.3	0.369
No. of peripheral tumors		13	100.00	26	86.21	0.229
No. of central tumors		0	0.00	3	13.79	0.229
Sequential CTx ^1^		10	76.9	11	37.9	0.019
Prior thoracic RT ^2^		7	53.8	8	27.6	0.101
Median time between ICB and RT (d)		8.5				
No. of pneumonitis		5	38.5	9	31.0	0.729
No. of pneumonitis grade 3		0	0.0	2	6.9	0.255
Median time until occurrence after RT (d)		71		47		0.174
**Dosimetric results**						
Pneumonitis V (cm^3^)	Mean	177.64		120.42		0.641
	SD ^3^	242.99		80.54		
	Median	68.82		99.43		
	Min ^4^	18.42		59.32		
	Max ^4^	603.04		313.59		
PTV ^5^ (cm^3^)	Mean	38.54		51.98		0.317
	SD	36.21		32.09		
	Median	26.30		46.70		
	Min	9.00		11.00		
	Max	146.00		323.00		
V20_EQD2_ (cm^3^)	Mean	29.62		75.21		0.039
	SD	28.21		45.18		
	Median	33.85		68.22		
	Min	4.23		10.63		
	Max	68.32		163.24		
EQD2_Pneumonitis_ (Gy)	Mean	37.50		62.72		0.317
	SD	45.23		28.60		
	Median	13.32		57.97		
	Min	0.27		10.29		
	Max	101.22		107.17		
V_pneumonitis_ EQD2 ≥ 20 Gy (%)	Mean	44.52		63.58		0.549
	SD	46.89		21.52		
	Median	22.26		71.14		
	Min	0.70		17.92		
	Max	99.28		91.78		
V_pneumonitis_ EQD2 < 10 Gy (%)	Mean	39.02		26.99		0.841
	SD	45.54		20.24		
	Median	19.39		20.68		
	Min	0.00		1.94		
	Max	98.96		68.11		
AUC_Pneumonitis_^6^	Mean	4012.69		6272.33		0.317
	SD	4284.33		2859.67		
	Median	1337.37		5799.10		
	Min	358.45		1028.48		
	Max	10,122.09		10,717.34		
MLD_total lung_ EQD2 (Gy)	Mean	6.43		7.33		0.641
	SD	4.28		3.22		
	Median	5.31		7.13		
	Min	2.03		1.34		
	Max	12.68		11.83		
V20_total lung_ EQD2 (%)	Mean	8.73		8.41		0.641
	SD	8.31		4.48		
	Median	5.86		7.07		
	Min	1.40		0.98		
	Max	22.50		15.09		

^1^ CTx means chemotherapy; ^2^ RT means radiotherapy; ^3^ SD means standard deviation; ^4^ Min and Max abbreviates minimum and maximum values; ^5^ PTV abbreviates planning target volume; ^6^ AUC stands for area under the curve of the dose volume histogram (DVH) of the pneumonitis volume.

## Data Availability

Data are contained within the article.
